# *Streptococcus pneumoniae* Serotype Epidemiology among PCV-10 Vaccinated and Unvaccinated Children at Gertrude’s Children’s Hospital, Nairobi County: A Cross-Sectional Study

**DOI:** 10.12688/f1000research.14387.2

**Published:** 2019-01-31

**Authors:** Michael Walekhwa, Margaret Muturi, Revathi Gunturu, Eucharia Kenya, Beatrice Kabera

**Affiliations:** 1 Department of Pathology, Gertrude's Children's Hospital, Nairobi, Kenya; 2 School of Medicine, Aga Khan University Hospital, Nairobi, Kenya; 3 Department of Biological Sciences, University of Embu, Embu, Kenya; 4 Department of Medicine, Kenyatta University, Nairobi, Kenya

**Keywords:** Streptococcus pneumoniae, serotypes, Nairobi, Quellung reaction, Optochin test, Bile solubility

## Abstract

**Background**: Serotype replacement and emergence of multidrug resistant *S. pneumoniae* has exacerbated the need for continuous regional serotype surveillance especially in the developing world. We investigated *S. pneumoniae* serotypes circulating among vaccinated and unvaccinated children ≤5 years in Nairobi County post PCV10 era.

**Methods**: A total of 206 vaccinated and unvaccinated children attending Gertrude’s Children’s Hospital (GCH) were recruited for this study. Nasopharyngeal swabs collected using Copan Flocked Swabs were the main study specimen. Culturing and isolation of *S. pneumoniae* was done on BA with gentamicin and BA plates respectively at the GCH main laboratory. Serotyping was done using the Quellung reaction at the KEMRI-Wellcome Trust, Kilifi.

**Results**: Out of the 206 subjects sampled, 20.39% (42) were found to be carriers of *S. pneumoniae*. About 52% (n=22) of the *S. pneumoniae* carriers had received the recommended dose of PCV-10, while 48% (n=20) of the carriers had not. Almost all (n=41; 19.90% of subjects) isolates contained non-vaccine type *S. pneumoniae* serotypes, while n=1 of the serotypes (in 0.49% of subjects) were untypeable. Serotypes 28F, 6A, 11A, 3 and 7C were prevalent in both vaccinated and unvaccinated children, whereas serotypes 23A, 17F, 35F, 48, 13 and 35B, and 23B, 20, 19B, 21, untypeable, 15B and 39 were found among unvaccinated and vaccinated groups, respectively.

**Conclusions**: All *S. pneumoniae* serotypes isolated from the subjects sampled were non PCV-10 vaccine type. These results therefore highlight the importance of monitoring and evaluation to provide epidemiological information to determine the effectiveness of PCV10 in Kenya’s Public health services.

## Introduction

*Streptococcus pneumoniae* is a friendly gram positive inhabitant of the human upper respiratory tract but can be highly invasive in some conditions ( Mitchell & Mitchell, 2010). It is a major cause of morbidity and mortality globally as it kills more children than any other illness ( Jones *et al*., 2010). *Streptococcus pneumoniae* is classified into serogroups (denoted by numbers and letters, e.g. 18c, 23f) ( Kellogg *et al*., 2001). There are over 90 known serotypes whose distribution and occurrence vary geographically across the globe ( Hackel *et al*., 2013). Different serotypes exhibit differing potentials to cause disease and may cause different syndromes in different age groups ( Harboe *et al*., 2009).

Some strains also have a greater potential to develop antibiotic resistance than others ( Song *et al*., 2012). The 13 most common serotypes of *S. pneumoniae* cause 80–93% of serious pneumococcal disease in children ( Johnson *et al*., 2010). According to the World Health Organization (WHO) and UNICEF Global Action Plan for the Prevention and Control of Pneumonia, pneumonia kills more children than any other illness in the world ( WHO & UNICEF, 2009). Given the high burden of under-five mortality associated with pneumonia, control efforts are critical to achieving Sustainable Development Goal 3 ( Colglazier, 2015). WHO and UNICEF estimates indicate that over 800,000 children under 5 years of age die from pneumococcal disease each year in the developing world ( O’Brien *et al*., 2009). In Kenya, an estimated one in every five children less than 5 years of age dies from this disease every year ( WHO, 2013).

*S. pneumoniae* vaccines protect against several severe forms of pneumococcal disease, such as meningitis, pneumonia and bacteremia ( Feldman & Anderson, 2014). These vaccines will not protect against these conditions if they are caused by agents other than *S. pneumoniae* or from strains not included in the vaccine ( Moffitt & Malley, 2011). The 10-valent pneumococcal conjugate vaccine (PCV10) was introduced into the Kenya Expanded Program on Immunization (KEPI) in February 2011 with a 2+1 schedule (at 6, 10, 14 weeks) without catch-up vaccinations ( Hammitt *et al*., 2014). The vaccine covers 1, 4, 5, 6b, 7f, 9V, 14, 18c, 19f and 23f serotypes.

Various *S. pneumoniae* serotypes with antigenic similarities are classified under the same groups (9A, 9L, 9N and 9V) while those lacking antigenic similarities are given numbers only (1, 2, 3, 4 and 5). The degree of interaction (cross-reactivity) between various *S. pneumoniae* groups may vary. For instance, serotypes 6A and 6B have identical chemical composition except for one of the bonds between two sugars yet they are highly cross-reactive but serotypes 19F and 19A are less reactive.

Pneumococcal conjugate (PCVs) and polysaccharide (PPVs) vaccines are designed according to their virulence mechanisms and how they generally interact with the human immune system ( Castañeda-Orjuela *et al*., 2012). The WHO has advised that all children ≤5 years should be immunized against pneumococcal disease and continuous surveillance done to keep out the disease especially in the developing world ( Vandenbos *et al*., 2013). The need for continuous surveillance has been exacerbated by the acute emergence of multi-drug resistant *S. pneumoniae* strains and escalated child mortality and morbidity due to pneumococcal disease, despite the availability of PCVs and PPVs ( Väkeväinen *et al*., 2010). This study therefore sought to establish the *S. pneumoniae* serotypes among vaccinated and unvaccinated children ≤5 years of age in Nairobi County, Kenya.

## Methods

### Study Location

This study was conducted among children ≤5 years attending the outpatient department of Gertrude's Children’s Hospital in Nairobi County between May 2017 and February 2018. Subjects were clinically assessed by a physician and those who presented with pneumococcal disease symptoms recommended to the study nurse for recruitment. Gertrude's Children’s Hospital is the largest standalone health care facility specializing in pediatric care in East and Central Africa. The hospital is accredited by the Joint Commission on International Accreditation (JCIA). *S. pneumoniae* isolation and stocking was done at Gertrude's Children’s Hospital Main Laboratory and capsular serotyping done at KEMRI Wellcome Trust, Kilifi, Kenya.

### Study Design

This was a descriptive cross-sectional study. *S. pneumoniae* serotype epidemiology among PCV-10 vaccinated and unvaccinated children between 6 months and 5 years of age was measured. Children who had no history of any chronic disease and whose parents or legal guardians consented to the study were systematically recruited. Children whose parents or legal guardians declined to give consent and those with any known immunosuppressive conditions were excluded from the study.

### Sample Size Determination

To determine the minimum sample size, the formula developed by Chow *et al.* (2007) was used, with a prevalence rate of 16% ( Agweyu *et al*., 2014).

$$n=\frac{z^{2}\;\hat{p}(1-\hat{p})}{m^{2}}$$

Where n= desired minimal sample size; *z*= standard normal deviation (1.96, from the tailed normal table); *p̂*= prevalence rate; and *m*= the desired degree of accuracy at a 95% confidence level of 0.05. This gave a sample size of 206.

### Identification of *S. pneumoniae*

Nasopharyngeal swabs were per nasally collected using Copan flocked swabs and temporarily suspended in Armies medium for transportation to the main laboratory. Each swab was inoculated onto a selective gentamicin with 5% sheep blood agar (BA) plate. All swabs were plated within 24 h of collection. The plates were incubated at 37°C in a 5% CO _2_ atmosphere and examined at 16–24 h and then again at 40–48 h for growth of *S. pneumoniae*. Isolates were identified as *S. pneumoniae* by colony morphology (Mucoid, draughtsman appearance, α-haemolysis) and susceptibility to optochin (positive, ≥14 mm zone of inhibition; negative, <14 mm zone of inhibition). Plates with colonies akin to *S. pneumoniae* morphological features but with optochin clearance zones below 14 mm were further subjected to solubility in bile salts (positive, bile soluble; negative, bile insoluble).

The isolation of a single colony indicated carriage. Single colonies were picked using sterile inoculating loops and evenly plated on BA. After 24–48 h, enough inoculum was stocked in brain heart infusion (BHI) agar with 5% sheep blood (Ultralab East Africa, Ltd), gently vortexed and stored at –70°C for serotyping.

### Serotyping of *S. pneumoniae*

Capsular serotyping was done using the Quellung reaction test. Frozen vials containing *S. pneumoniae* stocks stored at -70°C were thawed at room temperature for about 30 minutes. A loopful of the stored *S. pneumoniae* cells were suspended in 50 µl PBS and gently vortexed. Subsequently, 10 µl of the suspended cells were added on to a glass slide and mixed with 5 µl pooled antisera (Statens Serum Institute, cat. No.16744). The glass slide was swirled gently while observing for any agglutination reaction until a positive reaction was observed with various pooled antisera. The process was repeated with individual groups under various antisera pools.

After that, 10 µl of the suspended cells in PBS were added to a glass slide and mixed with various *S. pneumoniae* serotype-specific antisera included in the antisera pools that gave a positive reaction. This was done until a positive reaction with the particular serotype specific antisera was observed. Those serotypes that did not belong to any pool were typed directly until a positive agglutination reaction was observed. The cells/PBS/serotype-specific antisera mixture on the glass slide were covered with a cover slip and observed under a phase contrast microscope with a ×100 objective lens with oil emulsion.

## Results

Out of n=206 (100%) of the subjects sampled, n=97 (47.1%) were male and n=109 (52.9%) were female. In total, 68 (33.0%) of the children studied were within the age bracket of 6–12 months, 47 (22.8%) were between the ages of 13–24 months, 46 (22.3%) were between the ages of 25–36 months, 17 (8.3%) were between the ages of 37 and 48 months and 28 (13.6%) were between the ages of 49 and 60 months. Out of the total number of subjects (n=206) sampled, 20.39% (n=42) were found to be carriers of *S. pneumoniae*; 52% (n=22) of the *S. pneumoniae* carriers had received the recommended dose of PCV-10 immunization, while 48% (n=20) had not. All isolates (n=42; 20% of subjects) contained non-vaccine-type *S. pneumoniae* serotypes, while n=1 (0.49% of the subjects) of the serotypes were untypeable ( Table 1). In total, 18 different *S. pneumoniae* serotypes were found in this population. They include: 28F (8 instances), 6A (5 instances), 3 (4 instances), 23B (3 instances), 20 (3 instances), 23A (3 instances), 19B (2 instances), 17F (2 instances), 7C (2 instances), 11A (2 instances), 35F (1 instance), 15B (1 instance), untypeable (1 instance), 48 (1 instance), 35B (1 instance), 21 (1 instance), 39 (1 instance) and 13 (1 instance).

**Table 1.** Overall *Streptococcus pneumoniae* Carriage of Vaccine Type and Non-Vaccine Type Serotypes.

	All children		Vaccinated children		Unvaccinated children	
	N	%	N	%	N	%
Overall *S. pneumoniae* carriage	42	20.39	22	10.68	20	9.71
Proportion of *S. pneumoniae* Serotypes	count	%	count	%	count	%
PCV10	0	0.00	0	0.00	0	0.00
Non PCV10 serotypes	41	19.90	41	19.90	41	19.90
Non typeable	1	0.49	1	0.49	1	0.49

Various serotypes were found to be prevalent in different age groups. For instance, out of the 42 serotypes found, 9 (23.53%) were prevalent among children at 6–12 months of age (n=16). They include: 28F (4 instances), 11A (2 instances), 23A (2 instances), 3 (2 instances), 6A (2 instances), 17F (1 instance), 35F (1 instance), 7C (1 instance) and untypeable (1 instance). There were 7 (16.67%) serotypes prevalent among children at 13–24 months (n=8), including: 20 (2 instances), 21 (1 instance), 39 (1 instance), 28F (1 instance), 35B (1 instance), 17F (1 instance) and 13 (1 instance). There were 8 (19%) serotypes found among children of 25–36 months of age (n=12), including: 23B (3 instances), 19B (2 instances), 3 (2 instances), 20 (1 instance), 28F (1 instance), 7C (1 instance), 23A (1 instance) and 48 (1 instance). There were 3 (7%) serotypes prevalent among children at 37–48 months old (n=4), including: 6A (2 instances), 15B (1 instance) and 28F (1 instance).

**Figure 1.**
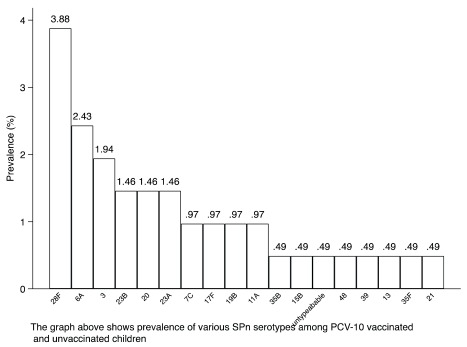
Percentage *S. pneumoniae* Serotype Distribution.

**Figure 2.**
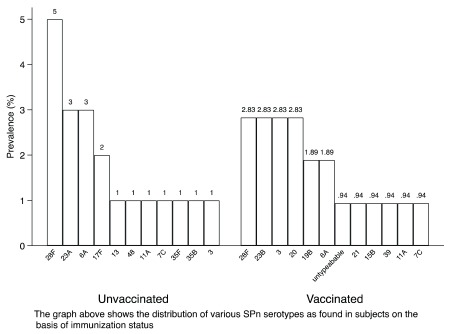
*S. pneumoniae* Serotype Distribution by PCV-10 Vaccination Status.

There were 2 (4.76% of the total) serotypes prevalent among children at 49–60 months (n=2): 6A (1 instance) and 28F (1 instance) ( Table 2). Out of the 42 isolates (found in 20.39% of subjects), serotype 28F was the most prevalent (3.88% of the total), followed by 6A (2.43%), 3 (1.94%) and 20, 23A and 23B all at 1.46% (n=3). Each of the serotypes 7C, 11A, 17F and 19B represented 0.97% (n=2) of the total serotypes, while serotypes: 13, 21, 39, untypeable, 48, 15B, 35B and 35F represented 0.49% (n=1) each of the total serotypes found ( Figure 1 and Figure 2). In total 51% (n=106) of the total sampled subjects were confirmed to have received a full dose of the PCV-10 vaccination as per the recommended schedule of immunization at 6, 10 and 14 weeks. Approximately 11% (n=12) of the immunized children were carriers of *S. pneumoniae* in their nasopharyngeal region; 10% (n=10) of the non-immunized group were also carriers ( Table 3). Serotypes 28F (5 instances), 23A (3 instances), 6A (3 instances), 17F (2 instances), 11A (1 instance), 3 (1 instance), 35F (1 instance), 48 (1 instance), 13 (1 instance), 35 (1 instance) and 7C (1 instance) were prevalent among the 9.71% (n=20) of the total sample group that had not received PCV-10 immunization. Serotypes 3 (3 instances), 28F (3 instances), 23B (3 instances), 20 (3 instances), 19B (2 instances), 6A (2 instances), 21 (1 instance), 11A (1 instance), 7C (1 instance), untypeable (1 instance), 15B (1 instance) and 39 (1 instance) were prevalent among the 10.68% (n=22) of the total sample group that received immunization ( Table 4).

**Table 2.** *S. pneumoniae* Serotype Distribution by Age.

	All	6–12 Months	13–24 Months	25–36 Months	37–48 Months	49–60 Months
Numbers with carriage (n)	42	16	8.00	12	4	2
Carriage (%)	20.39	23.53	17.02	26.09	23.53	7.14
Number of different serotypes seen	18	9	7	8	3	2
Serotypes seen	28F (8)	28F (4)	20 (2)	23B (3)	6A (2)	6A (1)
	6A (5)	11A (2)	21 (1)	19B (2)	15B (1)	28F (1)
	3 (4)	23A (2)	39 (1)	3 (2)	28F (1)	
	23B (3)	3 (2)	28F (1)	20 (1)		
	20 (3)	6A (2)	35B (1)	28F (1)		
	23A (3)	17F (1)	17F (1)	7C (1)		
	19B (2)	35F (1)	13 (1)	23A (1)		
	17F (2)	7C (1)		48 (1)		
	7C (2)	untypeable (1)				
	11A (2)					
	35F (1)					
	15B (1)					
	untypeable (1)					
	48 (1)					
	35B (1)					
	21 (1)					
	39 (1)					
	13 (1)					

*S. pneumoniae* serotypes as found in children at varying age groups

**Table 3.** *S. pneumoniae* Serotype Distribution by PCV-10 Vaccination Status.

Unvaccinated (100/206)			Vaccinated (106/206)		
Serotype	*n*	%	Serotype	*n*	%
28F	5	5	3	3	2.83
23A	3	3	28F	3	2.83
6A	3	3	23B	3	2.83
17F	2	2	20	3	2.83
11A	1	1	19B	2	1.89
3	1	1	6A	2	1.89
35F	1	1	21	1	0.94
48	1	1	11A	1	0.94
13	1	1	7C	1	0.94
35B	1	1	Untypeable	1	0.94
7C	1	1	15B	1	0.94
			39	1	0.94

**Table 4.** *S. pneumoniae* carriage by Vaccination Status.

Child Immunization Status	*S. pneumoniae*	*n*	%
PCV-10 Immunized	NGR	84	40.78
	*S. pneumoniae*	22	10.68
Non PCV10-Immunized	NGR	80	38.83
	*S. pneumoniae*	20	9.71
Total	N/A	206	N/A

NGR, no growth observed; SP *n*, *Streptococcus pneumoniae*; NA, not applicable

List of basic demographic information for each subject, with the size of the optochin clearance zone and serotype of Streptococcus pneumoniae, if found Click here for additional data file. Copyright: © 2019 Walekhwa M et al. 2019 Data associated with the article are available under the terms of the Creative Commons Zero "No rights reserved" data waiver (CC0 1.0 Public domain dedication).

## Discussion

This study found that 20.39% of all children studied, from both the PCV-10 vaccinated and unvaccinated groups, were carriers of *S. pneumoniae.* While this is a significant reduction from the pre-vaccine era, it is still high compared to malaria, diarrhea and HIV/AIDS ( Feikin *et al*., 2010). In total, n=41 of the serotypes found were non-vaccine type (in 19.90% of the subjects), with one additional untypeable serotype. This is a very important finding as it explains the high level of child morbidity and mortality due to pneumococcal disease despite the availability of PCV-10.

While these findings agree partially agree with those of ( Jacobs *et al.* (2008), where a significant decrease in the vaccine type *S. pneumoniae* serotypes found in isolates was observed, a 97.6% (n=41) decrease is, at the very least, surprising. This trend may be attributed to the increased level of antimicrobial misuse by a greater percent of the study population ( Domenech de Cellès *et al*., 2011). 10-valent pneumococcal conjugate vaccine contains 10 different serotypes, which include: 1, 4, 5, 6B, 7F, 9V, 14, 18C, 19F, 23F ( Slotved *et al*., 2016). None of these 10 serotypes was found in the study population yet this is the vaccine currently included in KEPI, targeting the same population.

*S. pneumoniae* carriage decreased with age as 11.65% (n=24) were obtained from children aged between 6–24 months and 8.74% (n=18) from children >24 months. The study results demonstrated a linear relationship between child age and *S. pneumoniae* carriage. A similar study done elsewhere reported findings that partly agree with this and partly disagrees ( Hill *et al*., 2008).

The former being attributable to development of *S. pneumoniae*-specific IgG antibodies due to vaccination and during that window before most children start attending school ( Corscadden *et al*., 2013). Unlike findings from a study by ( de Paz *et al.* (2015), serotype 28F was the most prevalent and was present in all five age groups profiled. This is a likely scenario of serotype replacement as *S. pneumoniae* attempts to evade the action of the immune system and eventually shares the resistant genes within the microbial community, especially in the nasopharyngeal region ( Donati *et al*., 2010).

Serotypes 28F, 6A, 11A, 3 and 7C were prevalent in both vaccinated and unvaccinated children, whereas serotypes 23A, 17F, 35F, 48, 13, 35B and 23B, 20, 19B, 21, untypeable, 15B, 39 were found among unvaccinated and vaccinated groups respectively. There exist different antigenic features between and within various strains of *S. pneumoniae* ( Song *et al*., 2012). While the majority, if not all, *S. pneumoniae* serotypes are capable of causing disease, the frequency with which they are isolated varies ( Kalin, 1998). In this case, vaccination would only be partially effective and, if so, due to inter-strain antigenic characteristics.

While trying to evade the action of the immune system, *S. pneumoniae* has a tendency to exchange resistant genes and other antigenic correlates at the nasopharyngeal region ( Johnston *et al*., 2014). Resistance to antimicrobial agents is occasioned by among other factors, misuse of antibiotics ( Dinsbach, 2012). This is largely due to lack of properly enforced antibiotic use regulations by the authorities.

## Data availability

The data referenced by this article are under copyright with the following copyright statement: Copyright: © 2019 Walekhwa M et al.

Data associated with the article are available under the terms of the Creative Commons Zero "No rights reserved" data waiver (CC0 1.0 Public domain dedication). 

**Dataset 1. List of basic demographic information for each subject, with the size of the optochin clearance zone and serotype of *Streptococcus pneumoniae*, if found.** DOI: http://doi.org/10.5256/f1000research.14387.d207458 ( Walekhwa *et al.*, 2018).
