# Supplementary material for: Streptococcus pneumoniae Serotype Epidemiology among PCV-10 Vaccinated and Unvaccinated Children at Gertrude’s Children’s Hospital, Nairobi County: A Cross-Sectional Study
Source: F1000Res. 2019 Jan 31;7:879. Originally published 2018 Jun 22. [Version 2] doi: 10.12688/f1000research.14387.2 (PMC6367659; doi:10.12688/f1000research.14387.2)
Supplement: Peer review history

# Peer review: Referee response for version 2

**DOI:** 10.5256/f1000research.18422.r43883

**Published:** 2019-02-07

**Authors:** Bartholomew N. Ondigo

**Affiliations:** 1 Centre for Global Health Research, Kenya Medical Research Institute, Kisumu, Kenya

Comments addressed.

I have read this submission. I believe that I have an appropriate level of expertise to confirm that it is of an acceptable scientific standard.

# Peer review: Referee response for version 1

**DOI:** 10.5256/f1000research.15656.r39482

**Published:** 2018-11-15

**Authors:** Felix Dube

**Affiliations:** 1 Division of Medical Microbiology, Department of Pathology, University of Cape Town, Cape Town, South Africa

The study reports pneumococcal carriage in a cohort of Kenyan children. This has significant implication when it comes to the evaluation of the impact of PCV10 on the population structure of pneumococcus.

 Specific comments:

1. Pneumococcus is a normal commensal, not "highly invasive" as is reported in intro.
2. The authors must avoid the use of non-standard nomenclature such as SPn. Further, there is inconsistent use of S. pneumoniae and pneumococcus throughout the text. These cannot be used interchangeably.
3. The introduction needs to be reworked and repetitions avoided, i.e. in paragraph 1 and 5, the authors talk about 90 serotypes. The last sentence of paragraph 5 does not include references.
4. The study was conducted between 2017 and 2018, it would really have benefited from the WHO working group report on pneumococcal carriage studies ^1^. Most importantly, 79% (164/206) being non-viable seriously indicates problems in the experimental design. The authors don't say anything about broth enrichment in order to improve recovery of the pneumococcus. Amies media is not ideal for pneumococcus compared to STGG. A 10ul innoculum is very little, did the authors attempt to use bigger volumes especially for the samples with no growth? A 2% BA plate is used as primary culture then selective media, if possible, do this in parallel.
5. Need to be more consistent in reporting proportions.
6. What was the PCV10 vaccine coverage at each timepoint? Also authors need to report non-pcv as non-PCV10 because some of the serotypes the report as "non-vacccine" such as serotype 3 are included in PCV13.
7. Repetition of results. The authors do not report any metadata looking at risk factors for carriage, hence the "epi" in title must fall out.
8. The authors repeatedly report "decrease" in carriage post PCV, but they really can’t say this without data on Pre-PCV10.
9. Avoid sweeping statement to infer serotype replacement if they actually don't show this evidence.
10. "While this is a significant reduction from the pre-vaccine era, it is still high compared to malaria, diarrhea and HIV/AIDS" doesn't make sense, what are the authors referring to?
11. "This is a very important finding as it explains the high level of child morbidity and mortality due to pneumococcal disease despite the availability of PCV-10." How do you arrive at this if working with carriage and not invasive disease isolates.
  This and other strong conclusions need to be avoided, you surely cant with 42 isolates.

I have read this submission. I believe that I have an appropriate level of expertise to state that I do not consider it to be of an acceptable scientific standard, for reasons outlined above.

## References

[1] SatzkeCTurnerPVirolainen-JulkunenAAdrianPVAntonioMHareKMHenao-RestrepoAMLeachAJKlugmanKPPorterBDSá-LeãoRScottJANohynekHO'BrienKLWHO Pneumococcal Carriage Working Group: Standard method for detecting upper respiratory carriage of Streptococcus pneumoniae: updated recommendations from the World Health Organization Pneumococcal Carriage Working Group. *Vaccine*.2013;32(1) : 10.1016/j.vaccine.2013.08.062 165-79 10.1016/j.vaccine.2013.08.062 24331112

# Peer review: Referee response for version 1

**DOI:** 10.5256/f1000research.15656.r39125

**Published:** 2018-11-01

**Authors:** Jackie K. Obey

**Affiliations:** 1 School of Health Sciences, Department of Medical Laboratory Sciences, University of Eastern Africa, Baraton, Eldoret, Kenya

The study carried out by the authors on *Streptococcus pneumoniae *is extremely important for Kenya. It addresses a major health problem that has been addressed by other authors in the past and for which a lasting solution is currently being sought. The study is detailed and was able to employ modern techniques to assess the carriage state of children at The Gertrude's Children Hospital, Nairobi, Kenya. The methods used were appropriate and in line with the study's objectives. The sample size was appropriately determined and descriptive statistics have been used appropriately to describe the results obtained by the authors.

 The title of the study however includes the word 'epidemiology' and this may lead the reader to think that the authors would have tried to determine factors that influenced the establishment of the research problem at the study site. The authors have not determined those factors or risks that lead to Kenyan children being vaccinated, yet not being protected by the PCV-10 vaccine. Those findings would then have given an idea of the risk of expose of children to *Streptococcus pneumoniae *disease and given the hospital and government the strategies to employ for preventive measures against the disease. The conclusion is brief and does not give recommendations to the Government of Kenya or The Gertrude's Children Hospital on what to do after the findings of the study.

I have read this submission. I believe that I have an appropriate level of expertise to confirm that it is of an acceptable scientific standard.

# Peer review: Referee response for version 1

**DOI:** 10.5256/f1000research.15656.r39126

**Published:** 2018-10-25

**Authors:** Bartholomew N. Ondigo

**Affiliations:** 1 Centre for Global Health Research, Kenya Medical Research Institute, Kisumu, Kenya

Title: To include Kenya

 Corresponding author contacts need to be indicated

 Abstract:

 Background:

 It needs to be indicated that circulating SPn serotypes are being investigated after the introduction of 10-valent pneumococcal conjugate vaccine (PCV10) in 2011.

 The methods need to be a summary of the techniques used in data collection. As it is written it appears to be copy pasted from the method section. Indicate number of children assessed. For instance:

 Materials:

 Two hundred and six children attending and not-attending in ……20-2009 were studied. Materials for study were pharyngeal swabs and sputum. Identification was performed using optochin disks, Quellung reaction,……agglutination on the glass, viewed under a phase contrast microscope and the sent to KEMRI- Kilifi for further confirmative identification tests.

 The conclusion seem alarming and need to suggest or indicate. Therefore Kenyan children receiving PCV-10 vaccine are not protected – Revise to something like:-

 This study highlights the importance of monitoring and evaluation to provide epidemiological information to determine the effectiveness of PCV10 in Kenya’s Public health services.

 Repeating ideas should be deleted - causing more deaths than any other infectious disease vs. kills more children than any other illness in the world.

 Introduction need to be shortened, preferably to three paragraphs.

 Methods:

 Were the children admitted or outpatient?

 All the source of equipment used and consumables need to be indicated, for instance incubator etc.

 The Research Ethics Committee that approved the study need to be indicated.

 Software used for calculation of %s need to be indicated?

 Results:

 Headings need to be introduced that are in agreement with the content. This will help the reader to when reading. Suggested possible headings include:

 Demography of the Study Participants

 Prevalence of SPn carriage status among PCV-10 vaccinated and unvaccinated children

 Prevalence of SPn carriage status by age – You probably have several age groups on this for instance  <1, 2 -4 years, 4 – 5 years.

 Authors need to clarify on the following:

 How many pneumococcus serotypes were identified? Which serotype was most frequent?

 Can you please arrange them in a descending order?

 Discussion:

 Adequate in content.

 Consider serotype replacement to enrich your discussion.

I have read this submission. I believe that I have an appropriate level of expertise to confirm that it is of an acceptable scientific standard, however I have significant reservations, as outlined above.

# Response

**Published:** 2019-01-06

**Authors:** Michael Walekhwa

**Affiliations:** Kenyatta University, Kenya

Dear Dr. Ondigo,

 Many thanks for your review of this article. 

 I have read through and keenly updated the areas you highlighted during your review.

 Kindly revisit.

 Many thanks
